# 1891. Descriptive Epidemiology of African-American Patients with Tuberculosis in in Detroit

**DOI:** 10.1093/ofid/ofad500.1719

**Published:** 2023-11-27

**Authors:** Avnish Sandhu, Erin Pollock, Shona Smith, James Sunstrum, Dana Kissner

**Affiliations:** Wayne State University , Detroit, Michigan; Wayne State University School of Medicine, Detroit, Michigan; Michigan Department Of Human Health Services, Detroit, Michigan; Michigan Dept. of Health and Human Services, Dearborn, MI; Wayne State University School of Medicine, Detroit, Michigan

## Abstract

**Background:**

Knowing local risk factors for tuberculosis (TB) facilitates identification of individuals with latent or active TB. The purpose of this study is to describe the epidemiology of TB among patients seen in the Detroit Health Department. We will refer to our study population as Detroit TB cases.

**Methods:**

A retrospective descriptive study was conducted in collaboration with the Michigan Department of Health and Human Services. TB data submitted to MDHHS from Detroit between January 2017 and December 2022 was evaluated. Information on demographics, genotype clusters, and clinical / lifestyle risk factors was collected

**Results:**

One-hundred forty-one TB cases were verified in Detroit during the study period and will be referred to as the study population. See Table 1 for baseline demographics. Sixty percent (85) of cases were males. Fifty-eight % (81) were Black / African American (B/AA). Sixty-two % (87) of cases were born in the U.S. Of persons with positive cultures for TB, 45% (37) were in a genotype cluster. Please see figure 1 for example of genotype cluster. The major clinical risk factors were diabetes mellitus in 31% (43) of cases, followed by incomplete treatment of latent TB infection (LTBI) in 11% (16). Major life-style risk factors included any substance use in 24% (34), non-injecting drug use in 18% (26), and excess alcohol use in 13% (18) of cases.

**Figure:**
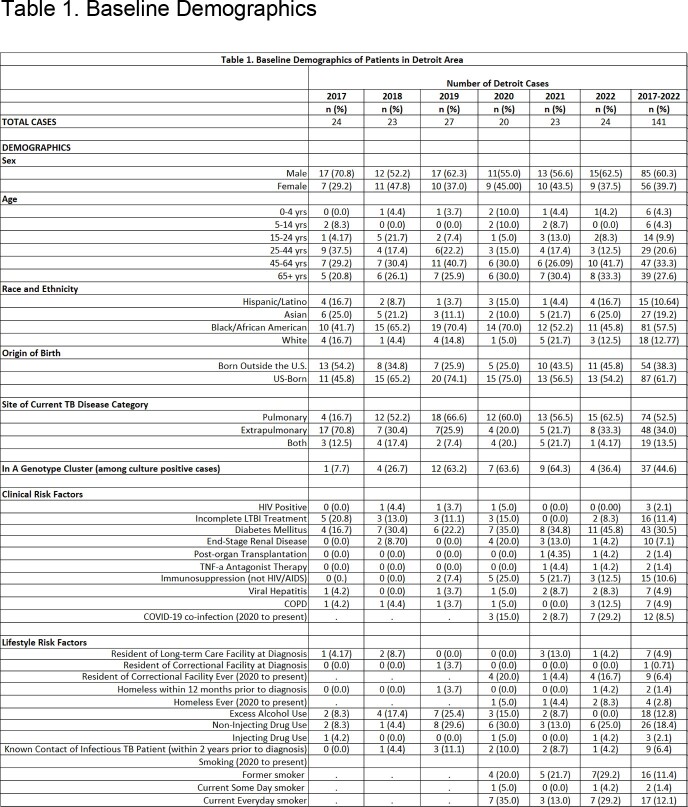

**Figure:**
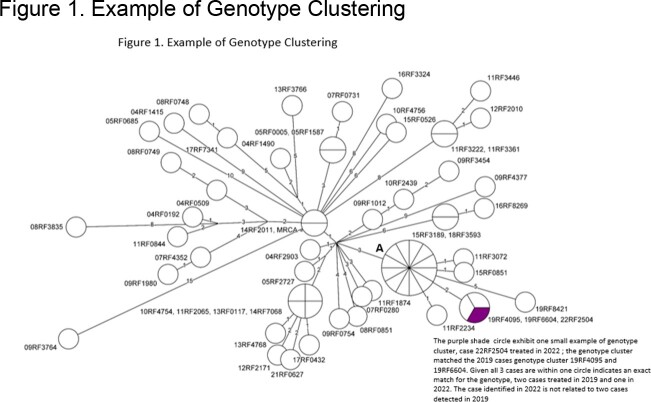

**Conclusion:**

This study highlights epidemiologic differences between individuals with TB in Detroit and the US. Almost 60% of the 141 Detroit TB cases were B/AA. Nationally, 10% of non-US-born individuals and 30% of US-born ones were B/AA in 2022. More than 60% of the Detroit cases were US-born. This is in stark contrast to the U.S., where 37% of cases in 2022 were US-born. A significant number of Detroiters belonged to a genotype cluster, indicating continued transmission of TB. Alcohol and drug use were major lifestyle risk factors, suggesting increased contact in shared venues contributing to transmission of TB. Knowing these local risk factors for TB should lead to earlier detection and treatment of TB and LTBI and eliminate opportunities for transmission.

**Disclosures:**

**All Authors**: No reported disclosures

